# Comparative study of gastric cancer and chronic gastritis via network analysis 

**Published:** 2018

**Authors:** Vahid Mansouri, Sina Rezaei Tavirani, Mohammad-Mehdi Zadeh-Esmaeel, Mohammad Rostami-Nejad, Mostafa Rezaei-Tavirani

**Affiliations:** 1 *Proteomics Research Center, Faculty of Paramedical Sciences, Shahid Beheshti University of Medical Sciences, Tehran, Iran*; 2 *Skin Research Center, Shahid Beheshti University of Medical Sciences, Tehran, Iran*; 3 *Gastroenterology and Liver Diseases Research Center, Research Institute for Gastroenterology and Liver Diseases, Shahid Beheshti University of Medical Sciences, Tehran, Iran *

**Keywords:** Chronic gastritis, Gene ontology, Biomarkers, Gastric cancer

## Abstract

**Aim:**

In this study the significant differentially expressed genes (DEGs) related to gastric cancer (GC) and chronic gastritis were screened to introduce common and distinctive genes between the two diseases.

**Background:**

Diagnosis of gastric cancer as a mortal disease and chronic gastritis the stomach disorder which can be considered as risk factor of GCs required safe and effective molecular biomarkers.

**Methods:**

Microarray profiles were downloaded from Gene Expression Omnibus (GEO) and analyzed via GEO2R. The candidate DEGs plus relevant genes from STRING database were interacted by Cytoscape software version 3.6.0 the central nodes were determined and analyzed.

**Results:**

JUN, GAPDH, FOS, TP53, PRDM10, VEGFA, and CREB1 as central nodes and TFF1 and ERG1 as the top changed expressed genes were determined as critical nodes related to gastric cancer. GAPDH, PRDM10, TP53, JUN, AKT1, EGFR, MAPK1, EGF, DECR1, and MYC were identified as common remarkable genes between GC and chronic gastritis.

**Conclusion:**

Identification of distinctive and common genes between GC and chronic gastritis can be useful in the early stage detection of disease and reducing risk of GCs.

## Introduction

 Gastric cancer (GC) is the third leading cause of cancer mortality in the world, especially in East Asia (1). Since GC biomarkers are not sufficiently sensitive and specific for diagnostic proposes endoscopy as an aggressive method is the common toll in diagnosis (2). Chronic gastritis the other stomach disorder is characterized by multistep, progressive, and life-long inflammation disease (3). Investigations indicated that there is correlation between gastritis and GCs (4). Early detection of gastric cancer is mostly depended to endoscopic methods (5). There are several documents about molecular mechanism of gastric cancer which emphasizes on the roles of numerous genes in onset and development of GC (6, 7). Genetics of chronic gastritis specially correlated with Helicobacter pylori revealed that different genes are involved in the growth of disease (8). 

Recently high throughput methods play a critical role to establish effective and nonaggressive diagnostic tools related to cancer diseases (9). Proteomic investigations and Protein-protein interaction (PPI) network are high throughput methods commonly used in clinical researches (10). Biomarkers such as Gastrokine-1, Antrum mucosal protein, Pepsinogen C, IPO-38 antigen are introduced as gastric cancer (6). However more investigations is required for a definitive proof of the introduction of disease biomarkers (11). Deregulation of IL-6 and TGF-β1 in chronic gastritis is investigated and confirmed (12, 13). Here microarray profiles of GCs and chronic gastritis patients versus the healthy samples are analyzed by network analysis to determined possible common and differential molecular features between the both diseases. 

## Methods

The microarray profiles of 10 healthy people versus 26 chronic gastritis and 35 gastric carcinomas (platform GPL2048) were downloaded from Gene Expression Omnibus (GEO). The profiles were analyzed via GEO2R and matched by box plot analysis. The 250 top significant differentially expressed genes (DEGs) were selected for each groups. Among 250 DEGs the genes that were characterized by fold change (FC) above 1.5 and P-value less than 0.05 were selected as significant genes related to chronic gastritis and gastric carcinomas. The top changed expressed genes were displayed as up and down regulated genes. The candidate DEGs plus 100 and 50 relevant genes from STRING database for chronic gastritis and gastric carcinomas respectively included to construct PPI network by Cytoscape software version 3.6.0 (14). The networks were analyzed by network analyzer plugin of Cytoscape. The networks were visualized and layout based on degree value. The top 10% of nodes based on degree value were identified as hub-nodes for the two diseases. In the similar way based on betweenness the bottleneck-nodes were determined for both diseases. The common hub and bottleneck nodes were introduced as hub-bottlenecks relative to the chronic gastritis and gastric carcinomas. The hubs, bottlenecks, and hub-bottlenecks were analyzed as central genes. For better understanding the common central genes between the both diseases were determined. Also the distinctive central nodes between two diseases were identified and discussed.

## Results

**Figure 1:**
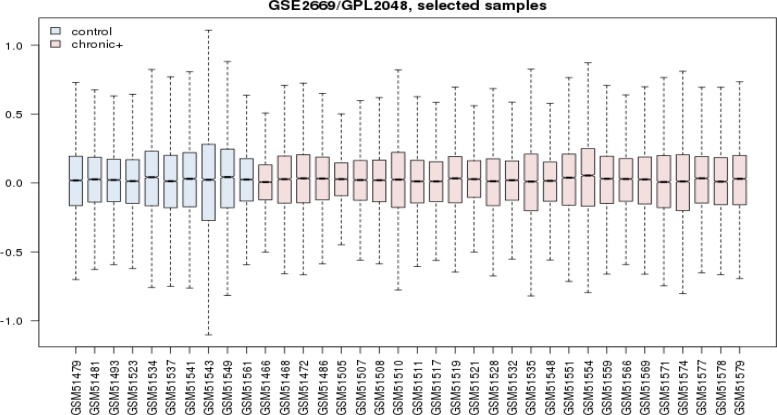
Box plot representation of gene expression profiles of 26 chronic gastritis patients versus 10 healthy samples are shown. Both groups are comparable

**Table 1:** The top 10% of nodes based on degree and betweenness centrality (BC) values were selected as hub and bottleneck genes respectively. The common hubs and bottlenecks were identified as hub-bottlenecks. The hub and bottleneck genes were colored red and yellow respectively and seven Hub-bottleneck genes are appeared as uncolored nodes. The nodes are sorted base on degree value

R	Display name	Description	Degree	BC
1	JUN	jun proto-oncogene	105	0.04
2	GAPDH	glyceraldehyde-3-phosphate dehydrogenase	100	0.03
3	FOS	FBJ murine osteosarcoma viral oncogene homolog	98	0.02
4	TP53	tumor protein p53	97	0.04
5	PRDM10	PR domain containing 10	91	0.02
6	MAPK1	mitogen-activated protein kinase 1	91	0.01
7	AKT1	v-akt murine thymoma viral oncogene homolog 1	90	0.01
8	VEGFA	vascular endothelial growth factor A	88	0.01
9	MYC	v-myc myelocytomatosis viral oncogene homolog (avian)	88	0.01
10	IL6	interleukin 6 (interferon, beta 2)	87	0.01
11	MAPK8	mitogen-activated protein kinase 8	87	0.01
12	CREB1	cAMP responsive element binding protein 1	86	0.01
13	EGFR	epidermal growth factor receptor	84	0.01
14	EGF	epidermal growth factor	84	0.01
15	CTNNB1	catenin (cadherin-associated protein), beta 1, 88kDa	65	0.01
16	DECR1	2,4-dienoyl CoA reductase 1, mitochondrial	65	0.02
17	AR	androgen receptor	63	0.01

**Figure 2:**
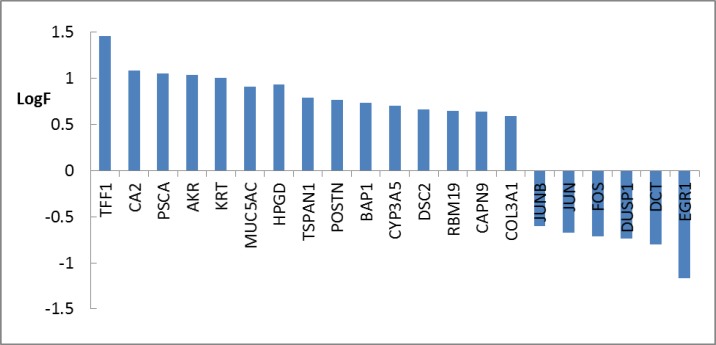
Numbers of 21 DEGs including 6 down-regulated and 15 up-regulated genes related to chronic gastritis are shown. Fold change above 1.5 was considered

**Figure 3:**
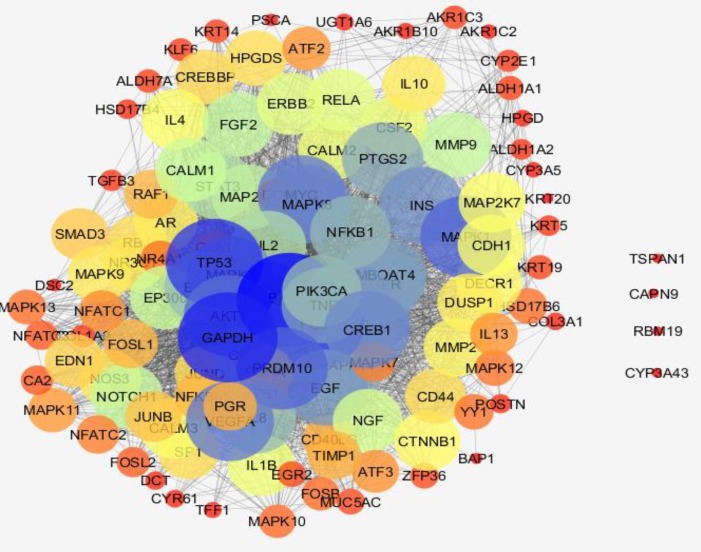
PPI network related to chronic gastritis is presented. The nodes are layout based on degree value. The node bigger size refers to larger amount of degree. Color from red to blue is corresponded to increment of degree

**Table 2:** The top 10% of nodes of gastric carcinoma based on degree and betweenness centrality values were selected as hub and bottleneck genes respectively. The common hub and bottleneck nodes were identified as a hub-bottlenecks. The hub and bottleneck genes were colored red and yellow respectively and nine Hub-bottleneck genes are appeared as uncolored nodes. The nodes are sorted based on degree value

R	Display name	Description	Degree	BC
1	GAPDH	glyceraldehyde-3-phosphate dehydrogenase	79	0.08
2	PRDM10	PR domain containing 10	70	0.05
3	AKT1	v-akt murine thymoma viral oncogene homolog 1	60	0.05
4	ALB	albumin	59	0.03
5	DECR1	2,4-dienoyl CoA reductase 1, mitochondrial	58	0.02
6	INS	insulin	57	0.05
7	TOP2A	topoisomerase (DNA) II alpha 170kDa	57	0.03
8	ACACA	acetyl-CoA carboxylase alpha	54	0.03
9	MAPK1	mitogen-activated protein kinase 1	53	0.02
10	ACACB	acetyl-CoA carboxylase beta	53	0.02
11	ACLY	ATP citrate lyase	52	0.02
12	CAT	catalase	52	0.01
13	SRC	v-src sarcoma (Schmidt-Ruppin A-2) viral oncogene homolog (avian)	52	0.02
14	TP53	tumor protein p53	51	0.02
15	EGFR	epidermal growth factor receptor	51	0.03
16	TXN	thioredoxin	51	0.03
17	HSP90AA1	heat shock protein 90kDa alpha (cytosolic), class A member 1	50	0.03
18	POTEF	POTE ankyrin domain family, member F	50	0.03
19	EGF	epidermal growth factor	49	0.03
20	JUN	jun proto-oncogene	48	0.03
21	GSR	glutathione reductase	45	0.07
22	MYC	v-myc myelocytomatosis viral oncogene homolog (avian)	44	0.03
23	UBA52	ubiquitin A-52 residue ribosomal protein fusion product 1	32	0.06

**Figure 4:**
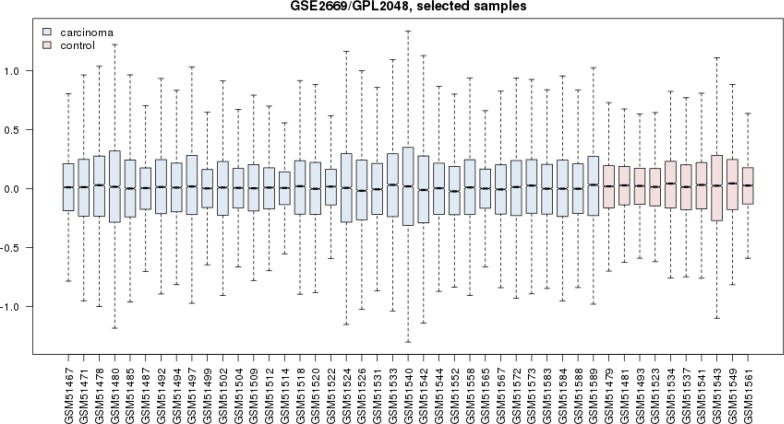
Box plot illustration of gene expression profiles of 35 gastric carcinoma patients versus 10 healthy samples are presented. Both groups are comparable

**Figure 5:**
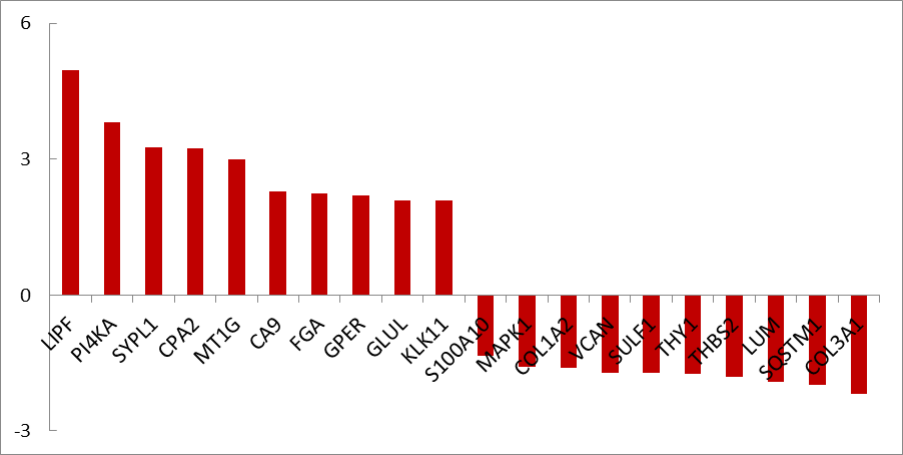
Numbers of 20 top DEGs including 10 top down-regulated and up-regulated genes related to gastric carcinoma are presented

**Figure 6:**
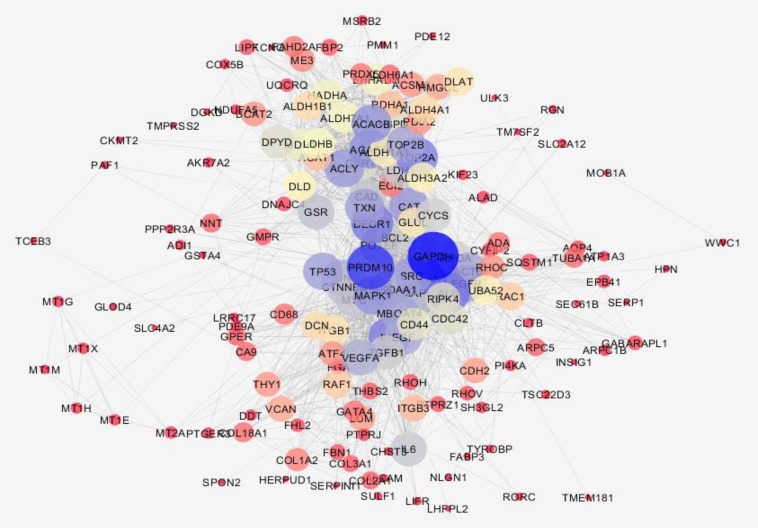
PPI network (The main connected component) related to gastric carcinoma is shown. The nodes are layout based on degree value. The node bigger size refers to larger amount of degree. Color from red to blue is corresponded to increase of degree

Gene expression profiles of 10 healthy samples and 26 patients were selected as control and chronic gastritis groups respectively. Box plot representation of gene expression profiles (figure 1) indicates that the two groups are comparable. Among the 250 top changed expression genes 26 significant DEGs were selected. Among the genes of a family (two family including AKR and KRT), the highest scored gene was nominated. Finally the 21 DEGs including 6 down-regulated and 15 up-regulated genes related to chronic gastritis are defined and displayed in the figure 2. The PPI network was constructed by the 26 significant DEGs and 100 added related genes. Except 3 genes the other were included in the network. The network includes 4 isolated nodes and a main connected component with 3498 edges (see figure 3). The central nodes including hubs, bottlenecks, and hub-bottlenecks were determined and shown in the table 1.

The gene expression profiles of gastric carcinoma group including 35 patients were compared with the profiles of healthy group (see figure 4). Based on box plot analysis the groups are analogous. The numbers of deregulated genes related to carcinoma (146 genes) was about 6 time greater than the DEGs of chronic gastritis. Therefore 10 top up and down-regulated DEGs related to carcinoma were selected and represented in the figure 5. The PPI network was constructed by 146 DEGs and additional 50 related genes. The network including 31 isolated genes and a main connected component (see figure 6) was analyzed and the central nodes (table 2) were determined. The common hubs, bottlenecks, and hub-bottlenecks between chronic gastritis and gastric carcinoma based were identified and tabulated in the table 3. The differential central nodes between the two diseases also were considered.

## Discussion

Disease gene expression profile can provide useful information about pathology and molecular mechanism of disorder (15). In figure 1 gene profiles of chronic gastritis patients are compared with the normal control samples. The mid of data are approximately equal. Hence the profiles are comparable. Among numerous DEGs there are 21 significant DEGs which are characterized by FC above 1.5. As it’s shown in figure 2 TFF1 and EGR1 are highly up and down regulated respectively. TFF1 is an important gene that plays a critical role in gastric glands differentiation (16). Investigation is shown that EGR1 is involved in early stage as well as progression of gastric cancer. However the role of EGR1 in inflammation and different cellular processes is reported and discussed (17). There is a question; which gene among the 21 DEGs plays the most important role in chronic gastritis. 

Based on gene expression quantity, TFF1 and EGR1 can be introduced as the most critical genes related to chronic gastritis. It’s well known that protein function and interactions are the two important correlated features of protein molecules that effect the vast varieties of biological processes in body (18). As it is shown in the figure 3 and table 1 the TFF1 and EGR1 are not included as central genes of chronic gastritis network but JUN is the top first central gene. This controversy may be due to large amounts of information about some proteins while the others are not well-known. For instance, there are 2,370,000 and 6640 documents about JUN gen and TFF1 gene respectively in google scholar. Based on this hypothesis, it is expected that EGR1 should be less known related to the JUN or GAPDH. The resulted search in google scholar about EGR1 and GAPDH are as 24,500 and 229,000 respectively. 

**Table 3:** The common hub-bottlenecks (H-Bs), hubs (12), and bottlenecks (Bs) between chronic gastritis and gastric carcinoma are presented as red color. The differential central nodes between the two studied diseases also are shown as uncolored genes

R	Gene name	Gastritis	Adenocarcinoma
1	GAPDH	H-B	H-B
2	PRDM10	H-B	H-B
3	TP53	H-B	H
4	JUN	H-B	B
5	AKT1	H	H-B
6	EGFR	B	H-B
7	MAPK1	H	H
8	EGF	B	B
9	DECR1	B	H
10	MYC	H	B
11	INS	-	H-B
12	TOP2A	-	H-B
13	ACACA	-	H-B
14	ALB	-	H-B
15	TXN	-	H-B
16	ACACB	-	H
17	ACLY	-	H
18	CAT	-	H
19	SRC	-	H
20	HSP90AA1	-	B
21	POTEF	-	B
22	GSR	-	B
23	UBA52	-	B
24	FOS	H-B	-
25	VEGFA	H-B	-
26	CREB1	H-B	-
27	IL6	H	-
28	MAPK8	H	-
29	CTNNB1	B	-
30	AR	B	-

The second reason may be related to the physical and chemical properties of the studied proteins. Therefore here it is recommended that both the top DEGs and top central nodes be considered as possible biomarker panel related to the disease. Validation of determined DEGs is an important methodological process which leads to screen the DEGs (19). The advantage of DEGs as biomarker is related to the suitable and significant change of their levels in body so they can be detected easy. However it is possible that the DEGs have not important role as drug target. If a DEG protein play role as central node it can be considered as crucial gene in onset and development of disease. It seems that JUN and FOS are the two critical genes related to the chronic gastritis. As it is depicted in the table 1 JUN is the first top central node and also FOS is determined as the third hub-bottleneck. These two crucial oncogenes are down-regulated (see figure 2) and may be common between chronic gastritis and gastric adenocarcinoma. In the similar way the DEGs and central nodes of gastric adenocarcinoma (see figures 4 - 6 and table 2) were identified. As mentioned in the result part the larger numbers of DEGs are related to gastric adenocarcinoma relative to chronic gastritis which refers to the complexity of cancer and vast verities of involved processes in adenocarcinoma. Gastric lipase (LIPF) is the top overexpressed gene that investigation was shown it is a highly specific genes related to stomach (20). As it is illustrated in the table 2 there are 9 hub-bottlenecks related to the gastric adenocarcinoma which 3 genes among them are involved in metabolic processes. Glyceraldehyde-3-phosphate dehydrogenase, insulin, and acetyl-CoA carboxylase alpha are the 3 genes which are responsible for carbohydrate and fatty acid metabolism (21, 22). The other central nodes are the genes that mostly involved in the cellular function and development. However albumin is the well-known carrier that is responsible for different function such as osmotic pressure regulation drug and hormone transport in body (23). Comparison of the central nodes of the networks of the both diseases (see table 3) revealed new glance about them. There are 10 common central genes including GAPDH and PRDM10 that play role as hub-bottlenecks in the two diseases. PRDM10 gene belongs to PRDM family that are responsible for cellular differentiation (24). TP53, JUN, and AKT1 are the 3 more important common central nodes between both diseases. INS, TOP2A, ACACA, ALB, and TXN are the key genes which play an important role in adenocarcinoma. INS & ACACA are well known lipid and carbohydrate related metabolite genes. However TOP2A & TXN are involved mostly in cellular process also as reported by Lim et al, TXN is introduced as biomarker of gastric cancer (25). As mentioned in table 3 FOS, VEGFA, & CREB1 are the critical genes involved in the chronic gastritis. Investigations indicates that CREB1 is involved in cancer cellular proliferation (26). Although FOS & VEGFA are known as vascular epithelial growth factors therefore they are cell growth factors (27). 

As we analyzed features of both diseases genetically, the common and differentially biomarker panels were determined for chronic gastritis and gastric adenocarcinoma. Our suggested markers can be used as diagnostic tools or drug target and also distinctive implements for both diseases.

It could be concluded that chronic gastritis and gastric adenocarcinoma can be differentiated based on molecular diagnosis. Also the common molecular pathological pathway between two diseases is arguable. Of course, this material requires more field research.
